# Optimizing transcutaneous spinal stimulation: excitability of evoked spinal reflexes is dependent on electrode montage

**DOI:** 10.1186/s12984-024-01524-5

**Published:** 2025-01-06

**Authors:** Kelly Lynn Thatcher, Karen Emily Nielsen, Evan Blake Sandler, Oliver John Daliet, Jennifer Ann Iddings, Edelle Carmen Field-Fote

**Affiliations:** 1 Hulse Spinal Cord Injury Research Lab, Shepherd Center, 2020 Peachtree Road NW, Atlanta, GA USA; 2 Department of Population Health Sciences, Georgia State University, 140 Decatur Street, Atlanta, GA USA; 3 Department of Applied Physiology, Georgia Institute of Technology, 555 14th Street NW, Atlanta, GA USA; 4 Department of Physical Therapy, Emory University, 1462 Clifton Road NE, Atlanta, GA USA

**Keywords:** Neuromodulation, Transcutaneous spinal stimulation, Spinal excitability, Electrodes

## Abstract

**Background:**

There is growing interest in use of transcutaneous spinal stimulation (TSS) for people with neurologic conditions both to augment volitional control (by facilitating motoneuron excitability), and to decrease spasticity (by activating inhibitory networks). Various electrode montages are used during TSS, with little understanding of how electrode position influences spinal circuit activation. We sought to identify the thoracolumbar electrode montage associated with the most robust activation of spinal circuits by comparing posterior root-muscle reflexes (PRM reflexes) elicited by 6 montages. Additionally, we assessed tolerability of the stimulation during PRM reflex testing.

**Methods:**

Fifteen adults with intact neurological systems participated in this randomized crossover study. PRM reflexes were evoked transcutaneously using electrode montages with dorsal–ventral (DV) or dorsal-midline (DM) current flow. DV montages included: [1] cathode over T11/T12, anodes over iliac crests (DV-I), [2] cathode over T11/T12, anodes over umbilicus (DV-U), [3] dual paraspinal cathodes at T11/12, anodes over iliac crests (DV-PI), and [4] dual paraspinal cathodes at T11/12, anodes over umbilicus (DV-PU). DM montages included: [5] cathode over T11/12, anode 5 cm caudal (DM-C), and [6] cathode over T11/12, anode 5 cm rostral (DM-R). PRM reflex recruitment curves were obtained in the soleus muscle of both lower extremities.

**Results:**

Lower reflex thresholds (mA) for dominant (D) and nondominant (ND) soleus muscles were elicited in DV-U (D: 46.7[33.9, 59.4], ND: 45.4[32.5, 58.2]) and DV-I (D: 48.1[35.3, 60.8], ND: 45.4[32.5, 58.2]) montages compared to DV-PU (D: 64.3[51.4, 77.1], ND:61.7[48.8, 74.6]), DV-PI (D:64.9[52.1, 77.7], ND:61.4[48.5, 75.5]), DM-C(D:60.0[46.9, 73.1], ND:63.6[50.8, 76.5]), and DM-R(D:63.1[50.3, 76.0], ND:62.6[49.8, 75.5]). DV-U and DV-I montages demonstrated larger recruitment curve area than other montages. There were no differences in response amplitude at 120% of RT(1.2xRT) or tolerability among montages.

**Conclusions:**

Differences in spinal circuit recruitment are reflected in the response amplitude of the PRM reflexes. DV-I and DV-U montages were associated with lower reflex thresholds, indicating that motor responses can be evoked with lower stimulation intensity. DV-I and DV-U montages therefore have the potential for lower and more tolerable interventional stimulation intensities. Our findings optimize electrode placement for interventional TSS and PRM reflex assessments.

*Clinical Trial Number:* NCT04243044.

**Supplementary Information:**

The online version contains supplementary material available at 10.1186/s12984-024-01524-5.

## Background

Transcutaneous spinal stimulation (TSS) is increasingly being utilized as a neurorehabilitation tool for improving walking function [1–4], spasticity [5, 6], and postural control [7] in people with neurologic conditions. TSS can be applied using clinically accessible devices, making it optimal for pairing with physical therapy interventions. While early studies have focused on people with spinal cord injury (SCI), emerging reports are investigating the effects of TSS in people with multiple sclerosis [8], stroke [9], traumatic brain injury [10], and cerebral palsy [11].

While frequently referred to as “spinal cord stimulation”, modeling studies indicate TSS activates large-diameter Ia afferents at the dorsal nerve roots, similar to epidural spinal stimulation [12, 13]. Hence, our use of the term transcutaneous spinal stimulation indicates electrode location and does not imply *direct* activation of spinal cord structures. Ia afferents synapse directly with spinal motoneurons, with subthreshold stimuli bringing motoneurons closer to activation threshold, and suprathreshold stimuli eliciting a monosynaptic reflex [12, 13]. TSS thereby improves motor output in persons with neurologic conditions by augmenting the motoneuronal excitability produced by volitional effort [7, 14, 15].

The same electrode positions used during interventional TSS have been used to measure spinal reflex excitability. Pulses of transcutaneous stimulation over the lumbosacral enlargement activate Ia afferents at the dorsal nerve roots and elicit short-latency evoked potentials, termed posterior root-muscle reflexes (PRM reflexes) [16]. Paired pulses confirm the reflex origin of the evoked responses, as depression of the second response confirms activation of primary afferent neurons (as opposed to direct activation of the anterior motor root) [13, 16]. PRM reflexes bear similarities to H-reflexes, the electrical analogue of the stretch reflex [16–18]. PRM reflexes demonstrate potential utility in measuring responses to neuromodulatory interventions that target Ia afferents, such as TSS, whole body vibration, and peripheral nerve stimulation.

A variety of electrode placements have been used for lower extremity PRM reflex testing and interventional TSS, with little understanding of which montage more efficaciously activates spinal circuitry. Reported cathode positions range from T9 to L2 including a single midline electrode over the interspinous space [1, 19], paired electrodes perpendicular to the spinal column [12, 16], or paired electrodes vertical to the spinal column [2, 4]. Reported anode positions include paired electrodes over the umbilicus [1, 20, 21], paired electrodes over the iliac crests [7, 22, 23], or a single electrode over the interspinous space rostral or caudal to the cathode [24]. Although some studies utilize multiple cathodes for interventional TSS, evidence supporting use of multiple cathodes is lacking. In fact, a recent study found force production was lower for plantarflexion and knee extension when using dual cathodes for TSS in comparison to a single cathode [25].

When considering electrode placement, it is important to appreciate electrode placement determines current flow, directly influencing activation of neural circuitry. A posterior cathode with anterior anode creates dorsal–ventral (DV) current flow, while a posterior cathode with posterior anode creates dorsal-midline (DM) current flow. Understanding differences in PRM reflex responses among different electrode montages is important for standardizing spinal reflex excitability measurement and optimizing interventional TSS. A limited number of studies have compared PRM reflex outcomes using different electrode positions [24, 26–29]. However, electrode montages commonly used in the SCI literature have not been systematically compared.

The goal of this study was to compare PRM reflexes of 6 commonly reported electrode montages in individuals with intact neurological systems. We aimed to identify montage(s) associated with the largest PRM reflex response at the lowest stimulation intensity and to assess stimulation tolerability among montages. Based on what is known about electrical current penetration, we hypothesized montages generating DV current flow would activate Ia afferents more efficaciously than montages generating DM current flow, as indicated by lower soleus reflex thresholds (RTs) and larger area under the PRM reflex recruitment curve (AUC). We additionally hypothesized montages with DM current flow would be better tolerated than DV current flow due to the absence of abdominal contractions generated by anode placement.

## Methods

This study was carried out with approval of the Shepherd Center Research Review Committee. All participants gave written informed consent prior to study enrollment in accordance with the Declaration of Helsinki. This study was funded by National Institutes of Health grant R01HD101812 (ECF-F) and the Hulse SCI Research Fund. The funders played no role in the design, conduct, or reporting of this study.

### Participants

Individuals who met the following inclusion criteria were eligible for study participation: ≥ 18 years of age, no changes in prescription medication use over the prior 2 weeks, ability and willingness to authorize use of protected health information, ability to follow multiple directions, and ability to communicate pain/discomfort. Individuals were excluded from study participation if they had any of the following exclusion criteria: history of neurologic injury/disease, or cardiovascular irregularities, current pregnancy, implanted stimulators, or skin lesions, irregularities, or sensitivities.

### Electrode montages

**Fig. 1:**
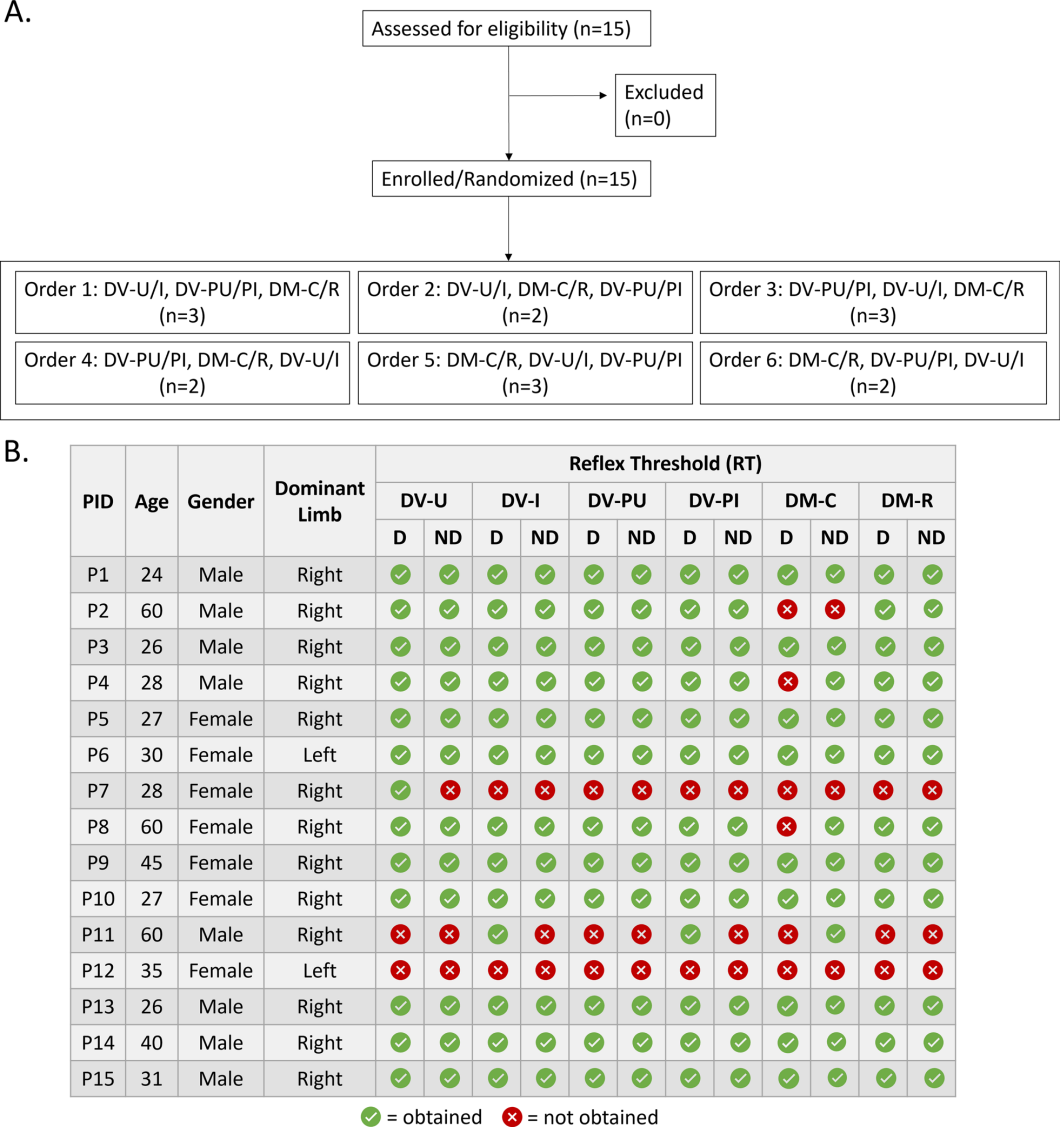
**A** Consort diagram outlining participant enrollment and randomization order. In this cross-over design, participants were randomized into groups dictating the order they received six electrode montages. All participants received all six montages across three sessions. **B** Participant demographics and reflex thresholds obtained per individual in each montage

**Fig. 2:**
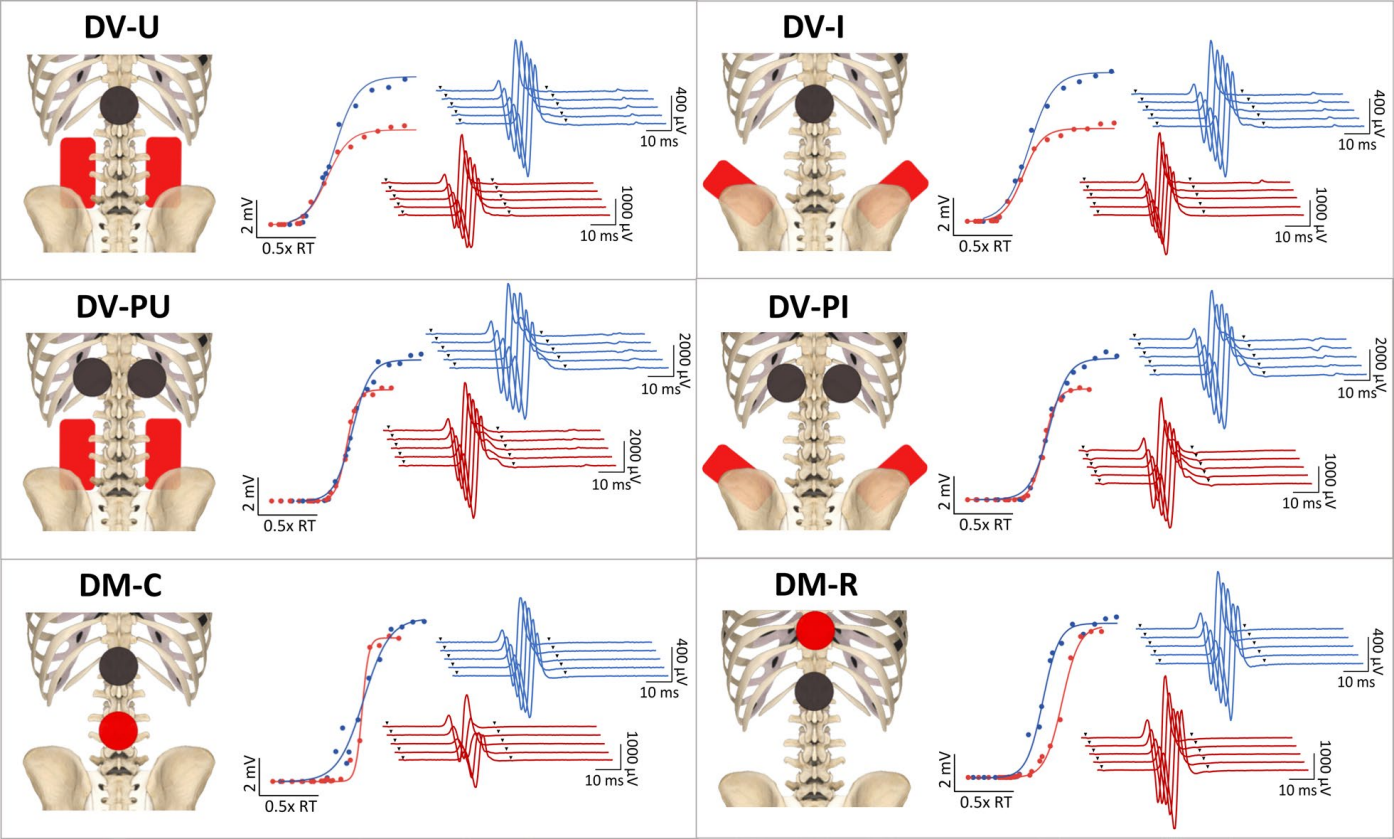
Cathode (black) and anode (red) positions for 6 electrode montages (left) with associated recruitment curves (center) and posterior root-muscle reflex response traces elicited by a stimulation intensity of 1.2xRT (right) from a representative participant (P13). Dorsal–ventral umbilicus (DV-U): cathode over T11/T12 with anodes over the umbilicus; dorsal–ventral iliac crests (DV-I): cathode over T11/T12 with anodes over iliac crests; dorsal–ventral paraspinal umbilicus (DV-PU): paraspinal cathodes at T11/12 with anodes over the umbilicus; dorsal–ventral paraspinal iliac crests (DV-PI): paraspinal cathodes at T11/12 with anodes over iliac crests; dorsal-midline caudal (DM-C): cathode over T11/12 with an anode 5 cm caudal; and dorsal-midline rostral (DM-R): cathode over T11/12 with an anode 5 cm rostral. Blue = dominant soleus muscle, red = nondominant soleus muscle, inverted triangles = time of stimulus application. Note the different scales for response traces across montages

Utilizing a randomized, crossover design, six electrode montages were tested in pairs over three sessions. Electrode montages evaluated in this study were selected based on their use in recent SCI literature by investigators with strong citation metrics and named based on expected current flow [1, 6, 7, 15, 17, 22, 24, 30]. Order of montage testing was randomized both between and within sessions. Participant allocation to each montage order is depicted in the CONSORT diagram (Fig. 1A). Sessions were separated by ≥ 24 h to prevent potential carryover effects of repeated electrical stimulation. Four DV and two DM montages were evaluated (Fig. 2). DV montages included [1] dorsal–ventral iliac crests (DV-I): cathode over T11/T12 and anodes over iliac crests, [2] dorsal–ventral umbilicus (DV-U): cathode over T11/T12 and anodes over umbilicus, [3] dorsal–ventral paraspinal iliac crests (DV-PI): paraspinal cathodes at T11/12 and anodes over iliac crests, and [4] dorsal–ventral paraspinal umbilicus (DV-PU): paraspinal cathodes at T11/12 and anodes over umbilicus. DM montages included [5] dorsal-midline caudal (DM-C): cathode over T11/12 and anode 5 cm caudal, and [6] dorsal-midline rostral (DM-R): cathode over T11/12 and anode 5 cm rostral. For DV montages, cathode(s) were 5 cm round electrodes and anodes were 9 × 5 cm rectangular interconnected electrodes. For DM montages, 5 cm round electrodes were used for cathode and anode. For umbilical montages, anodes were placed 5 cm apart on either side of the umbilicus. For iliac crest montages, anodes were placed with superior border oriented laterally and inferior border oriented medially. Manual palpation of spinous processes by a physical therapist (author KLT/EBS) was used to determine cathode placement. Skin under the cathode was swabbed with isopropyl alcohol and abraded (NuPrep, Weaver and Company, Aurora, CO) to decrease impedance. Conductive gel (Spectra 360, Parker Laboratories, Fairfield, NJ) was placed around the border of the cathode(s) to reduce risk of skin irritation at the interface between skin tissue and electrode edge. To ensure consistent stimulating electrode placement across sessions, cathode location and referencing anatomical landmarks (i.e. inferior scapular boarder) were marked on transparent film and used for reference in subsequent sessions.

### PRM reflexes

Electromyographic activity (EMG) was measured in the soleus muscle bilaterally using pre-amplified surface EMG electrodes (Motion Lab Systems, Baton Rouge, LA). Prior to EMG electrode placement, the skin over the soleus was swabbed with isopropyl alcohol and abraded to decrease impedance. To ensure consistent EMG electrode placement across sessions, EMG electrode location and referencing anatomical landmarks (i.e. patella and tibial tuberosity) were marked on stockinette sleeves placed on each lower extremity. EMG signals were acquired (MA300, Motion Lab Systems, Baton Rouge, LA), digitized (Power 1401, Cambridge Electronic Design, Cambridge, UK), and recorded at a sampling rate of 2 kHz for offline analysis [17, 31, 32] using sweep-based data capture and analysis software (Signal, Cambridge Electronic Design, Cambridge, UK).

Data were acquired with the participant lying supine. Pillows supported the participant’s head and knees as needed for comfort. As position influences PRM reflex responses [33], position was determined during the first session and remained consistent across subsequent sessions within each participant. PRM reflexes were elicited by monophasic, rectangular stimulation pulses with a 1 ms pulse width [34] using a constant current stimulator (Digitimer DS7AH, Hertforshire, UK). Paired stimulation pulses (40 ms inter-pulse interval) [35] were delivered (Grass S88X, Natus Neurology, Middleton, WI) with a minimum of 7 s between pulse pairs [24]. Depression of the second response was determined by calculating the difference between response amplitudes for the first and second stimuli and dividing by the response amplitude of the first stimulus. When acquiring PRM reflex recruitment curves, pulses were delivered starting at a stimulation intensity of 10 mA, increasing in increments of 10 mA until 30 mA, followed by increments of 5 mA. RT was defined as the stimulation intensity required to elicit a peak-to-peak PRM reflex response amplitude of ≥ 100 µV in at least 50% of trials [20] in each soleus muscle independently. When finding RT, stimulation intensity was increased in increments of 1 mA. PRM reflex recruitment curves were collected beginning at a subthreshold stimulation intensity (≤ 30 mA, dependent upon RT). Stimulation intensity was increased in increments of 5 mA until the soleus response plateaued or a stimulation intensity of 100 mA was achieved. A maximum stimulation intensity of 100 mA was imposed to avoid acute discomfort accompanying higher stimulation intensities. In addition to the 5 mA increments in stimulation intensity, PRM reflexes were collected at the stimulation intensity equivalent to 120% of RT (1.2xRT) if 1.2xRT fell below 100 mA. Three or five stimuli were repeated at each stimulation intensity for subthreshold responses and responses ≥ RT, respectively. Participants reported stimulation tolerability for each montage on a 0–10 visual analog scale with 0 indicating “absolutely tolerable” and 10 indicating “not at all tolerable.” The tolerability rating was obtained after completing the full recruitment curve, and therefore represents an average rating across all stimulation intensities. Sensation descriptors contributing to the participant’s tolerability rating were recorded.

### Extremity dominance

The dominant lower extremity was determined by the following question taken from the Waterloo Footedness Questionnaire-Revised, “If you were asked to shoot a ball on target, which leg would you use to shoot the ball?” [36].

### Data processing

**Fig. 3:**
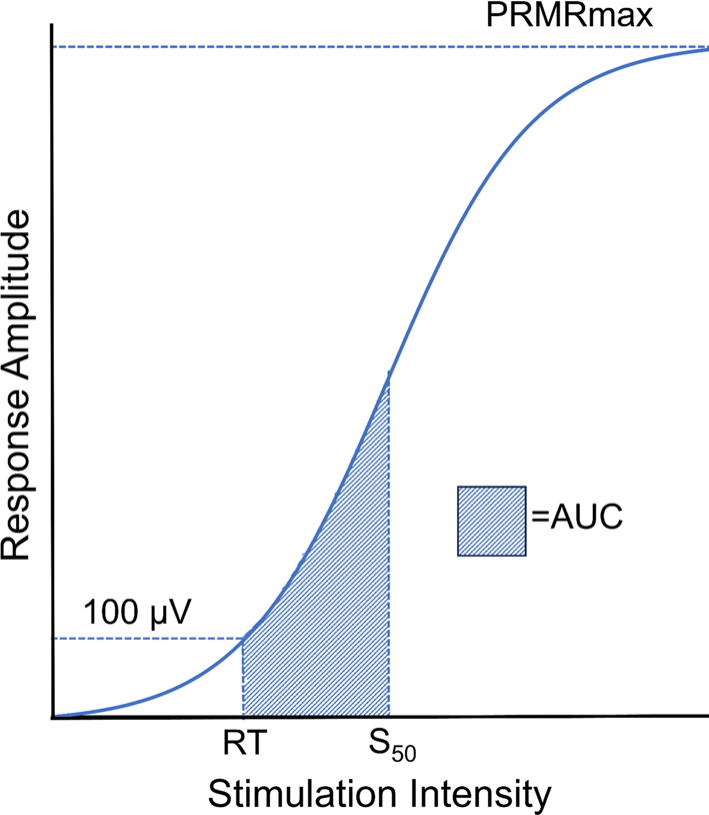
Model posterior root-muscle reflex recruitment curve with labeled outcomes. Values acquired during data collection include RT. Values derived from curve fitting include S_50_, AUC, and PRMRmax. Reflex threshold (RT): stimulation intensity required to elicit a reflex response > 100 µV in at least 50% of trials; S_50_: stimulation intensity required to elicit a response that is 50% of maximum posterior root muscle reflex amplitude (PRMRmax); Area under the curve (AUC): integrated recruitment curve area from RT to S_50_

Peak-to-peak soleus PRM reflex response amplitudes were exported and processed using custom MATLAB codes (MathWorks, Inc., Natick, MA). For all participants, soleus recruitment curves were generated for the dominant and nondominant soleus muscle for each montage by averaging PRM reflex response amplitudes for each stimulation intensity tested. Within each recruitment curve, stimulation intensity, *s*, was normalized to the acquired RT (i.e. *s*/RT) to account for inter-individual variability. Using a modified Boltzmann equation, non-linear curve fitting was performed for all recruitment curves for which both RT and 1.2xRT were obtained:

$$PRMR\left( s \right) = \frac{{PRMR_{max} }}{{1 + e^{{m\left( {S_{50} - s} \right)}} }} ,$$

where PRMR_max_ is the maximum response amplitude estimated by the function, S_50_ is the stimulation intensity required to elicit a response amplitude 50% of PRMR_max_, and m is the slope parameter of the Boltzmann function (Fig. 3) [31, 37]. The Levenberg–Marquardt algorithm (lsqcurvefit, Optimization Toolbox, The MathWorks, Natick, MA) was used to solve non-linear least-squares curve fitting. Initial guess inputs for the Levenberg–Marquardt algorithm were calculated as follows: PRMR_max_ – maximum average response amplitude acquired during data collection; S_50_ – mean normalized stimulation intensity averaged from the nearest data point above and below the value of 50% of PRMR_max_; *m* – slope of a linear regression fitted to the data points from RT to 1.2xRT, inclusive. Default termination criteria for the optimization function were used (600 for the maximum number of function evaluations and 1e^−6^ for function tolerance). Area under the recruitment curve (AUC) was calculated by numerically integrating the optimized Boltzmann equation from RT to S_50_.

### Statistical analysis

Statistical analyses were completed in R (R Foundation for Statistical Computing, Vienna, Austria). Outcomes were visualized and descriptive statistics calculated to check model assumptions and identify potential outliers. For each outcome, multilevel models with random intercept for participant and fixed effects for montage, lower extremity, and interaction between montage and lower extremity were the starting point for analyses, with modifications described below for specific tests. Pairwise comparisons were calculated as differences in estimated marginal means using the Tukey method for p-value adjustment for multiple testing and Kenward-Roger degrees of freedom following estimation of the multilevel model.

To assess the proportion of variability in each outcome explained by the participant for each montage, we used ICC model ICC (1,1). We selected this model as it does not include lower extremity in the underlying multilevel model, thereby reflecting an assumption that the dominant and nondominant lower extremities are interchangeable.

## Results

Fifteen adults with intact neurological systems, aged 24–60 years, participated in this study (Fig. 1B). Of the 180 recruitment curves collected (15 participants × 2 lower extremities × 6 montages), RT and 1.2xRT were not acquired for 36 and 10 curves respectively, as acquiring these data would have required us to exceed our a priori stimulation limit of 100 mA. Because both RT and 1.2xRT were required for Boltzmann curve fitting, data from 134 recruitment curves were included in final analyses. For all study outcomes, estimated marginal means and confidence intervals are reported in Table 1.

**Table 1:** Marginal mean values followed by model-based 95% confidence intervals [lower limit, upper limit] for dominant (D) and nondominant (ND) lower extremities for reflex threshold (RT), response amplitude (RA) at 1.2xRT, area under the curve (AUC) from RT to S50, 1.2xRT, and S50

		DV-U	DV-I	DV-PU	DV-PI	DM-C	DM-R
RT (mA)	D	46.7 [33.9, 59.4]	48.1 [35.3, 60.8]	64.3 [51.4, 77.1]	64.9 [52.1, 77.7]	60.0 [46.9, 73.1]	63.1 [50.3, 76.0]
	ND	45.4 [32.5, 58.2]	45.4 [32.5, 58.2]	61.7 [48.8, 74.6]	61.4 [48.5, 75.5]	63.6 [50.8, 76.5]	62.6 [49.8, 75.5]
RA @ 1.2xRT (µV)	D	2344 [1192, 3497]	2826 [1622, 4029]	2856 [1651, 4061]	2983 [1806, 4160]	2429 [1191, 3666]	2554 [1378, 3730]
	ND	1632 [456, 2808]	1545 [368, 2722]	1791 [614, 2969]	1607 [430, 2784]	1893 [697, 3089]	2629 [1476, 3781]
AUC (µVxRT)	D	422 [275, 568]	382 [235, 528]	266 [116, 416]	264 [114, 414]	319 [160, 477]	276 [126, 426]
	ND	484 [334, 633]	518 [372, 665]	329 [179, 479]	278 [128, 428]	347 [188, 505]	250 [103, 396]
1.2xRT (mA)	D	49.1 [38.5, 59.8]	49.8 [39.1, 60.5]	68.7 [58.0, 79.4]	68.4 [57.7, 79.1]	67.0 [56.3, 77.7]	68.2 [57.6, 78.9]
	ND	49.6 [39.0, 60.2]	50.2 [39.5, 60.9]	69.2 [58.5, 79.9]	68.9 [58.2, 79.5]	67.4 [56.7, 78.1]	68.7 [58.1, 79.3]
S50 (mA)	D	52.8 [42.1, 63.4]	53.2 [42.5, 63.8]	71.5 [60.8, 82.1]	72.2 [61.5, 82.9]	70.1 [59.2, 80.9]	69.9 [59.2, 80.5]
	ND	53.2 [42.6, 63.9]	53.6 [43.0, 64.2]	71.9 [61.3, 82.6]	72.6 [62.0, 83.3]	70.5 [59.7, 81.3]	70.3 [59.7, 81.0]

### Confirmation of reflex responses

Depression of the second stimulus response of the PRM reflex was used to confirm the evoked response was due to afferent fiber activation [13, 16]. The response amplitude corresponding to the second stimulus of the paired pulse was lower than the first response in 98.75% of RT trials and 99.21% of 1.2xRT trials.

### Reflex Threshold (RT)

RT indicates the minimum stimulation intensity required to elicit a reflex response. Due to the a priori stimulation intensity maximum of 100 mA, complete RT data (all 6 montages and both lower extremities) were acquired for 9 participants, partial RT data were acquired for 5 participants, and no RT data was obtained for 1 participant (P12). The DM-C montage had the lowest RT acquisition rate (Fig. 1B). Sensitivity analyses were undertaken to explore potential impacts of missing values on RT, including comparison of the lower extremity and montage of missing compared to non-missing RT, repeating analyses with values of 100 mA and 120 mA substituted for missing RT values, and repeating analyses excluding participants with partially missing RT. All analyses resulted in findings consistent with the following results.

**Fig. 4:**
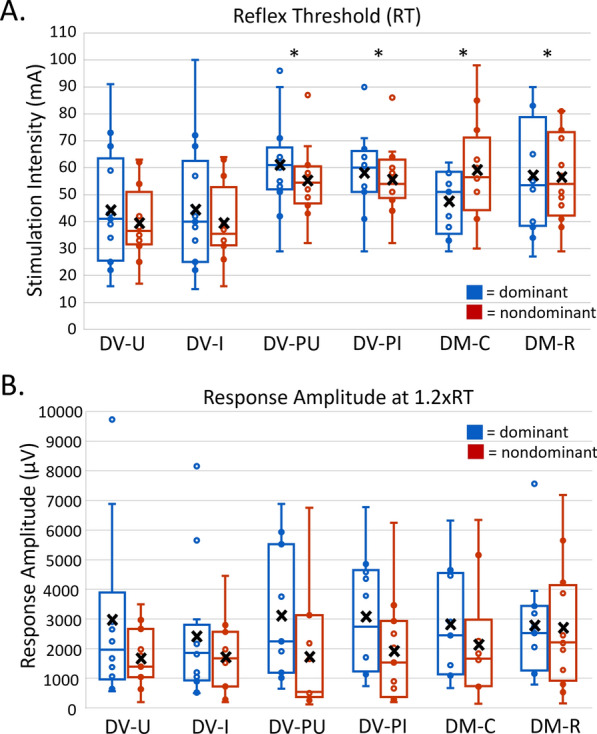
Recruitment curve outcomes in dominant (blue) and nondominant (red) soleus muscles. * = p < 0.05; X = mean. For reflex threshold (A), DV-U was significantly lower than DV-PU, DV-PI, DM-C, and DM-R for both dominant and nondominant soleus muscles. DV-I was significantly lower than DV-PU, DV-PI, DM-C, and DM-R for both dominant and nondominant soleus muscles. For response amplitude at 1.2xRT (B), there were no significant differences across montages for either dominant or nondominant soleus muscles

Significant differences in RT were identified across montages (F (5, 117.92) = 23.95, p < 0.001). DV-U and DV-I demonstrated significantly lower soleus RT (mA) in dominant and nondominant lower extremities compared to other montages (Fig. 4A). Mean soleus RT for DV-U and DV-I were not statistically different (D: 1.39, p = 1.00; ND:0, p = 1.00). No significant differences in soleus RT were identified between dominant and nondominant lower extremities (F(1, 117.87) = 0.67, p = 0.42).

### Response amplitude at 1.2 × reflex threshold (1.2xRT):

Response amplitude at 1.2xRT reflects output of spinal circuitry within the ascending portion of the recruitment curve. Due to the stimulation intensity maximum of 100 mA, complete response amplitude at 1.2xRT data were acquired for 6 participants, partial data were acquired for 7 participants, and no data was obtained for 1 participant (P12). There were no significant differences in peak-to-peak response amplitude at 1.2xRT among montages (F(5, 106.33) = 0.51, p = 0.77). Significant differences in response amplitude at 1.2xRT were identified between dominant and nondominant soleus muscles (F(1, 106.09) = 11.73, p < 0.001), with dominant response amplitudes typically exceeding nondominant response amplitudes. On average, responses in the dominant soleus muscle were 144% larger compared to the nondominant soleus muscle (Fig. 4B).

### Area under the curve (AUC)

**Fig. 5:**
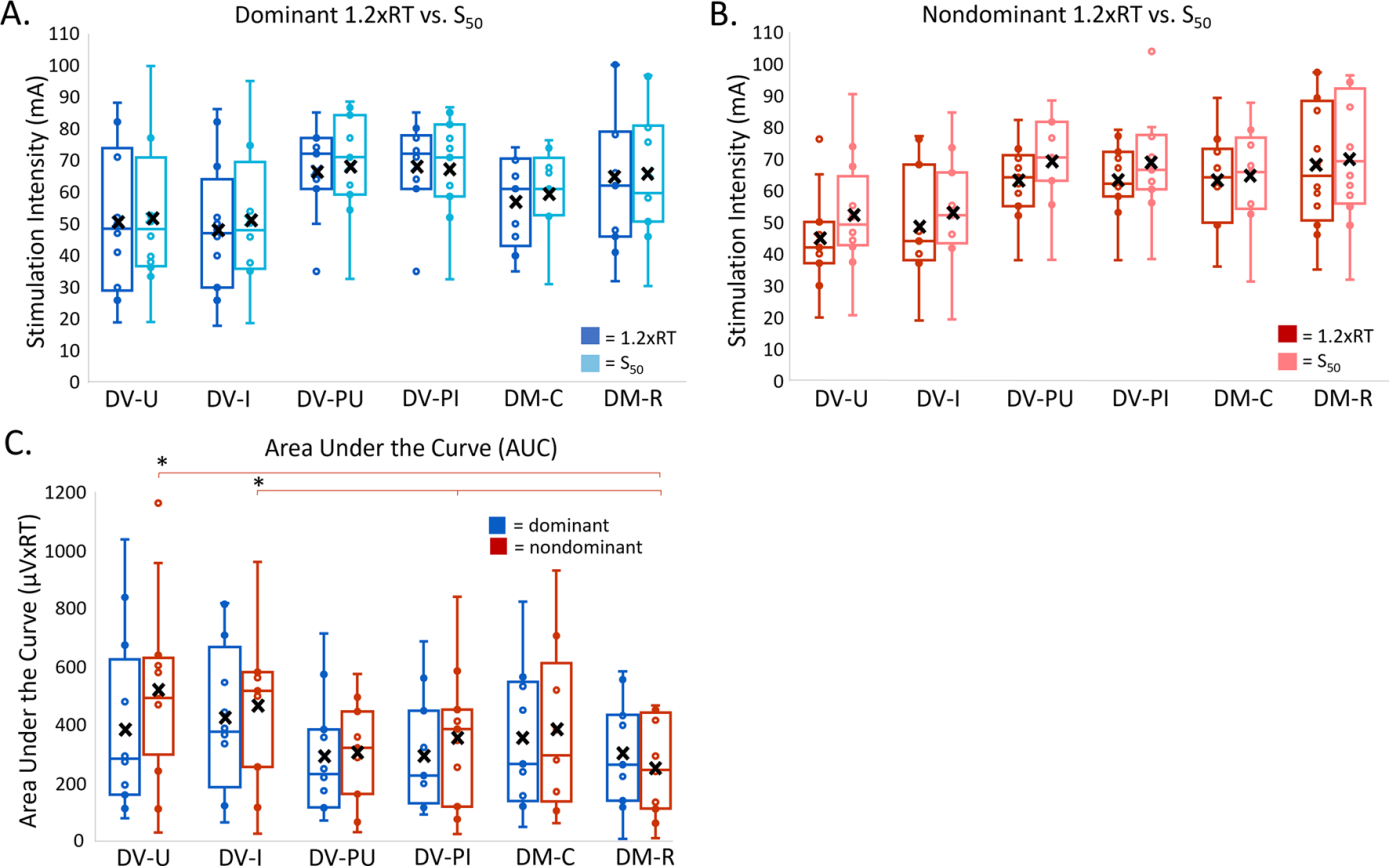
Recruitment curve outcomes in dominant and nondominant soleus muscles. * = p < 0.05; X = mean. There are no significant differences between 1.2xRT and S50 in the dominant (**A**) and nondominant (**B**) soleus muscles. AUC (**C**) was generally higher in DV-U and DV-I montages with significance found only in the nondominant soleus muscle where DV-U was significantly larger than DM-R and DV-I significantly larger than DM-R and DV-PI

The total output of activated spinal circuits can be assessed by calculating AUC. We calculated AUC between RT and S_50_ because this range is representative of stimulation intensities most commonly used for interventional TSS [7, 15]. Due to stimulation intensity maximum of 100 mA, complete AUC data were acquired for 8 participants, partial data were acquired for 4 participants, and no data was obtained for 1 participant (P12). There were significant differences in AUC among montages (F (5, 108.99) = 5.24, p < 0.001). Overall, AUC values were larger for DV-U and DV-I compared to other montages (Fig. 5C). Significant differences in AUC among montages were identified in the nondominant soleus muscle only, with AUC for DV-U significantly larger than DM-R (233.69, p = 0.03, 95%CI 15.7–451.7), and DV-I significantly larger than DM-R (268.46, p = 0.01, 95%CI 55.7–481.3) and DV-PI (240.46, p = 0.02, 95%CI 22.2–458.8). There was no significant difference in AUC between dominant and nondominant soleus muscles (F(1, 108.60) = 2.14, p = 0.15).

### Stimulation intensity at S_50_ compared to 1.2xRT

S_50_ is the stimulation intensity required to elicit a response that is 50% of PRMR_max_. S_50_ represents the point of the recruitment curve with the steepest slope and, therefore, has the greatest sensitivity to change when used as a measurement. While calculation of S_50_ requires completion of a full recruitment curve and curve fitting, 1.2xRT can be calculated based on a partial recruitment curve and could serve as a proxy to S_50_. Due to the stimulation intensity maximum of 100 mA, complete data for S_50_ were derived for 8 participants, partial data were derived for 4 participants, and no data was obtained for 1 participant (P12). As with 1.2xRT, on average, S_50_ was lower in DV-U and DV-I compared to other montages (Table 1). When comparing the stimulation intensities of 1.2xRT and S_50_ within montages, post-hoc tests did not identify any significant differences between these two measures in either soleus muscle (Fig. 5A, B).

### Tolerability

Twelve participants (P4-P15) provided tolerability ratings. Differences in tolerability ratings among montages were not significant (F (5, 55) = 1.13, p = 0.35). The estimated marginal mean tolerability rating and corresponding 95% CI per montage were as follows, DV-U: 4.17 [2.78, 5.55], DV-I: 4.33 [3.10, 5.57], DV-PU: 3.92 [2.97, 4.87], DV-PI: 3.75 [2.54, 4.96], DM-C: 4.92 [3.38, 6.45], DM-R: 4.17 [2.72, 5.61]. “Muscle contraction” and “sharpness” were the most commonly reported sensory descriptors contributing to higher tolerability ratings.

### Intra-individual variability

Separating observations by soleus muscle (dominant or nondominant) and response amplitude (RT or 1.2xRT), we computed intra-individual means (iMeans) and intra-individual standard deviations (iSDs) for peak-to-peak response amplitudes for each montage. We calculated proportional iSDs (iSD/iMean) to produce a single value reflecting the intra-individual variability for each montage and soleus muscle. Proportional iSDs were generally lower for 1.2xRT as compared to RT. Separate analyses for RT and 1.2xRT found no differences in proportional iSD among montages (RT: F(5, 121.82) = 0.62, p = 0.68; 1.2xRT: F(5, 103.71) = 0.42, p = 0.83). Proportional iSD was lower for the nondominant soleus muscle at RT (F(1, 121.45) = 6.76, p = 0.01, estimated difference = 0.08, 95% CI 0.02–0.15), but generally lower for the dominant soleus muscle at 1.2xRT (F(1, 102.8) = 4.16, p = 0.04, estimated difference = 0.03, 95% CI 0.00–0.06).

## Discussion

Neuromodulatory interventions can be a valuable adjuvant to physical therapy. The activation of Ia afferents can both increase motoneuron excitability to augment volitional activation [21] and activate inhibitory circuits to decrease reflex excitability [38]. For this reason, it is valuable for physical therapists to know what electrode montages best activate these neural structures. We found DV-U and DV-I montages were associated with significantly lower soleus RT in both dominant and nondominant lower extremities compared to other montages, indicating activation of spinal circuitry was achieved at lower stimulation intensities using these montages. DV-U and DV-I montages also had larger AUCs, with significant differences among montages observed only in the nondominant soleus muscle. This indicates greater overall activation of spinal circuits across stimulation intensities between RT and S_50_ with these two montages compared to the other montages evaluated.

TSS is typically delivered at stimulation intensities near RT [6, 19]. Given DV-U and DV-I montages demonstrated lower RTs, these montages may require lower stimulation intensities to activate neural structures targeted during interventional TSS. Lower stimulation intensities decrease risk for skin burns and improve tolerability. Our results differ from a previous study that found no significant differences between montages analogous to DV-U and DM-R [24]. A more recent study found montages using two cathodes at midline and a montage using a rectangular cathode at midline had lower RT values than a montage analogous to DV-I [28]. We used conventional electrophysiologic analyses to compare the TSS montages most commonly used in the SCI literature, some differences identified with other montages could be attributable to differences in methodology. For example, while most studies construct recruitment curves based on response amplitude at a specified stimulation intensity, both of the aforementioned studies constructed recruitment curves based on area under the full-wave rectified waveform. Also, while most studies base RT on values that have been acquired through direct measurement, both of these studies used RT values derived from curve fitting. Finally, while most investigators define RT based on the stimulation intensity required to elicit a response of a pre-specified amplitude, both of these studies used a nonstandard definition of RT using calculated values based on slope of the recruitment curve [24, 28]. Therefore, methodological variations may account for differences in results between previous studies and this study.

At 1.2xRT, there were no significant differences in peak-to-peak response amplitude among montages. While some investigators have analyzed differences in response amplitudes at PRMRmax between montages [24, 28], there has been no prior comparison of response amplitude at a normalized stimulation intensity on the ascending portion of the curve. Interventional TSS commonly uses stimulation intensities below those required to elicit maximum responses. For this reason, it is important to understand responses elicited at intensities used in clinical application. It is not fully understood why the dominant soleus muscle has larger response amplitudes; however, we propose that neuroplastic mechanisms contribute to increased excitability of spinal circuits that control the dominant lower extremity.

AUC provides insight into the overall output of neural circuits for a given individual. DV-U and DV-I montages have larger AUCs in comparison to other montages, indicating recruitment of more neural structures at stimulation intensities between RT and S_50_. To our knowledge, this is the first study to analyze differences in AUC among montages. When stimulation is delivered concurrently with volitional effort, intensities at and above RT have been demonstrated to increase muscle activation in persons with neurological injury [7]. Greater recruitment of neural structures by TSS, as denoted by larger AUC, may lead to enhanced rehabilitation outcomes.

S_50_ is another commonly used measure of neural excitability, including motor evoked potentials from transcranial magnetic stimulation and H-reflex testing [38–40]. Stimulating at an intensity corresponding to S_50_, the point with greatest potential for modulation, allows for greater sensitivity to identify change due to an intervention. Within a specific montage, when we compared values for 1.2xRT versus S_50_, there were no significant differences found between these measures. This finding suggests these two measures could be used interchangeably when using PRM reflexes to measure changes in spinal reflex excitability. Using 1.2xRT as a proxy for S_50_ would make it possible to measure changes in PRM reflexes following an intervention without the need to obtain a full recruitment curve.

We hypothesized DM montages may be more tolerable due to absence of abdominal contractions. However, our results demonstrated no significant differences in tolerability rating among montages. Given DV-U and DV-I demonstrate similar electrophysiological outcomes, participant preference may be used to determine anode placement between these two options [32, 41].

Based on our findings that DV-U and DV-I montages demonstrated lower RTs and larger AUCs, we recommend utilizing DV-U or DV-I montages in the clinical setting to optimize Ia afferent recruitment during interventional TSS and PRM reflex assessments for the soleus. Given the minimal differences between DV-U and DV-I montages, we recommend using participant discretion based on sensory tolerance to determine anode placement over the umbilicus or iliac crests. Our participants had no history of neurological injury and PRM reflexes were elicited under the same conditions; thus, we conclude the observed results reflect differences in recruitment of neural structures, particularly afferent fibers at the dorsal nerve roots. Future studies should assess the impact of different montages when paired with physical therapy interventions.

## Conclusions

While the stimulation devices used in this study were not clinical devices, interventional TSS can be applied with meaningful effects using clinically accessible stimulators [1, 5, 30]. TSS has also been used to modulate spinal reflex excitability and reduce spasticity based on activation of spinal inhibitory circuits [5, 6], with one study using stimulators commonly available in the clinic [5]. Generally, lower frequencies (e.g., 30 Hz) are used for motor activation while higher frequencies (e.g., 50 Hz) are used to reduce spasticity. Some devices that are not yet clinically available use a high frequency carrier wave, which is believed to improve comfort of the stimulation. However, this claim has been questioned, with evidence that high frequency carrier waves are associated with lower levels of neural activation, leading to less motor activation along with perception of greater comfort [42, 43].

Since the effects of TSS are based on activation of peripheral sensory fibers, it bears similarity to other forms of afferent input used for neuromodulation, such as vibration and peripheral nerve somatosensory stimulation [44–46]. In addition to the activation of spinal circuits, stimulation of peripheral sensory afferents has been shown to influence the excitability of cortical circuits and to promote adaptive neuroplasticity when combined with training [46–48]. The advantage of TSS is that a single cathodal electrode can activate multiple spinal roots, while peripheral somatosensory nerve stimulation is more selective by virtue of electrode location.

## Limitations

Our study focused on comparing montage differences in the soleus muscle as this muscle is important for functional outcomes, is frequently involved in spastic responses during daily life activities (i.e., clonus), and is often used in the literature as a target muscle for assessment of spinal reflex excitability [49]. Future studies should investigate the influence of electrode montages, and electrode size, on other muscles that can be targeted through TSS. Our study acquired PRM reflexes up to 100 mA to avoid acute discomfort; therefore, our results cannot be applied to individuals whose RT falls above 100 mA. Although this study included only individuals with intact neurological systems, the dorsal nerve roots and their central connections, which influences PRM reflex acquisition, remain intact below the level of lesion in individuals with SCI. Therefore, we predict our findings of montage differences will apply to people with SCI. TSS targets 1a afferents at the dorsal nerve roots, which remain intact after SCI. However, there are changes in the excitability of spinal circuits after SCI that could possibly influence responses, and therefore results from neurologically intact individuals may not be fully generalizable to individuals with neurologic conditions. Although maximum stimulation intensities differed between participants, tolerability ratings did not differ. Since stimulation during PRM reflex assessments involved paired pulses separated by at least 7 s compared to the continuous stimulation typically applied during intervention, our results may not have a direct correlation with tolerability to interventional TSS. Additionally, the tolerability of a monophasic waveform may be different than a biphasic waveform, most commonly used for interventional TSS.

## Supplementary Information

Supplementary material 1.

## Data Availability

Datasets are available upon request to the corresponding author.

## Abbreviations

TSS: Transcutaneous spinal stimulation
SCI: Spinal cord injury
PRM reflexes: Posterior root-muscle reflexes
DV: Dorsal–Ventral
DM: Dorsal-Midline
RT: Reflex Threshold
DV-I: Dorsal–Ventral Iliac Crests
DV-U: Dorsal–Ventral Umbilicus
DV-PI: Dorsal–Ventral Paravertebral Iliac Crests
DV-PU: Dorsal–Ventral Paravertebral Umbilicus
DM-C: Dorsal–Ventral Caudal
DM-R: Dorsal–Ventral Rostral
EMG: Electromyographic
PRMRmax: Posterior root-muscle reflex maximum response amplitude of the recruitment curve
AUC: Area Under the Recruitment Curve
iMean: Intra-Individual Means
iSD: Intra-Individual Standard Deviations
