# Supplementary material for: Optimizing transcutaneous spinal stimulation: excitability of evoked spinal reflexes is dependent on electrode montage
Source: J Neuroeng Rehabil. 2025 Jan 6;22:2. doi: 10.1186/s12984-024-01524-5 (PMC11702053; doi:10.1186/s12984-024-01524-5)
Supplement: Supplementary file 1 — Supplementary material 1. [file 12984_2024_1524_MOESM1_ESM.docx]

|  |  | DV-U | DV-I | DV-PU | DV-PI | DM-C | DM-R |
| --- | --- | --- | --- | --- | --- | --- | --- |
| RT (mA) | D | 46.7 [33.9, 59.4] | 48.1 [35.3, 60.8] | 64.3 [51.4, 77.1] | 64.9 [52.1, 77.7] | 60.0 [46.9, 73.1] | 63.1 [50.3, 76.0] |
|  | ND | 45.4 [32.5, 58.2] | 45.4 [32.5, 58.2] | 61.7 [48.8, 74.6] | 61.4 [48.5, 75.5] | 63.6 [50.8, 76.5] | 62.6 [49.8, 75.5] |
| RA @ 1.2xRT (µV) | D | 2344 [1192, 3497] | 2826 [1622, 4029] | 2856 [1651, 4061] | 2983 [1806, 4160] | 2429 [1191, 3666] | 2554 [1378, 3730] |
|  | ND | 1632 [456, 2808] | 1545 [368, 2722] | 1791 [614, 2969] | 1607 [430, 2784] | 1893 [697, 3089] | 2629 [1476, 3781] |
| AUC (µVxRT) | D | 422 [275, 568] | 382 [235, 528] | 266 [116, 416] | 264 [114, 414] | 319 [160, 477] | 276 [126, 426] |
|  | ND | 484 [334, 633] | 518 [372, 665] | 329 [179, 479] | 278 [128, 428] | 347 [188, 505] | 250 [103, 396] |
| 1.2xRT (mA) | D | 49.1 [38.5, 59.8] | 49.8 [39.1, 60.5] | 68.7 [58.0, 79.4] | 68.4 [57.7, 79.1] | 67.0 [56.3, 77.7] | 68.2 [57.6, 78.9] |
|  | ND | 49.6 [39.0, 60.2] | 50.2 [39.5, 60.9] | 69.2 [58.5, 79.9] | 68.9 [58.2, 79.5] | 67.4 [56.7, 78.1] | 68.7 [58.1, 79.3] |
| S50 (mA) | D | 52.8 [42.1, 63.4] | 53.2 [42.5, 63.8] | 71.5 [60.8, 82.1] | 72.2 [61.5, 82.9] | 70.1 [59.2, 80.9] | 69.9 [59.2, 80.5] |
|  | ND | 53.2 [42.6, 63.9] | 53.6 [43.0, 64.2] | 71.9 [61.3, 82.6] | 72.6 [62.0, 83.3] | 70.5 [59.7, 81.3] | 70.3 [59.7, 81.0] |

*Table 1*
